# Molecular Docking and Analysis of Survivin Delta-Ex3 Isoform Protein

**DOI:** 10.2174/1874104500802010016

**Published:** 2008-03-27

**Authors:** Z Ezziane

**Affiliations:** Department of Information Technology, Higher Colleges of Technology, Al Ain Women’s College, Al Ain, P.O. Box 17258, United Arab Emirates

## Abstract

This project explores molecular models of Survivin Delta-Ex3, H-Ras, and their binding sites, and generates energy optimized 3D coordinates of docked poses and conformations of the XY2 ligand molecule in the active site of Delta-Ex3. The aim is to propose an effective anti-cancer drug that induces apoptosis and inhibits tumor angiogenesis.

## INTRODUCTION

Wikberg and co-workers [1] are applying proteochemometrics as a bioinformatics entity to conduct biomedical research and drug design. This newly developed technology for functional genomics analysis has the potential to be applied over entire genomes and proteomes. Molecular modeling and chemoinformatics represent a crucial factor of drug discovery in every pharmaceutical industry. Rational drug design methods accelerate the process by speeding up the discovery of new chemical entities that may become new drugs. Structure-based design shows precisely the location and orientation of bound inhibitors and describes the physicochemical properties.

Survivin is a baculovirus inhibitor of apoptosis (IAP) repeat (BIR) motif protein and has essential roles in mitosis and cell cleavage. When overexpressed as it is in cancer cells, survivin is present in interphase during which is reported to suppress caspase-3 activity. It is expressed generally during normal embryonic development, but only in a small subset of adult normal differentiated tissues, including the colonic epithelium, uterine endometrium, vascular endothelium, and the subventricular region of normal brain [2-5].

It was reported that survivin’s function in cancer is mainly as an IAP, blocking mitochondrial-dependent apoptosis [6, 7]. However, survivin is also reported later to have a role in a mitotic checkpoint as a chromosomal passenger protein [8]. This family of proteins aligns the chromosomes properly during mitosis and maintains accurate cell division in normal cells [9]. It also avoids the development of abnormal numbers of chromosomes that may occur during the transition from a nonmalignant to a malignant phenotype [10].

There are several survivin isoforms including 2B, 3B, 2alpha, and Delta-Ex3 [11]. These proteins are directly involved in apoptosis. Delta-Ex3 is also found to have a role in angiogenesis [12].

Ras is a small GTPase (regulatory hydrolase) wherein healthy cells shuttles between on and off states — Activated (RAS-GTP) by guanine exchange factors (GEFs) and inactivated (RAS-GDP) by RasGAP [13]. Ras proteins are essential for proliferation, cell adhesion, apoptosis, and cell migration. When Ras is not properly regulated, it also plays a role in proliferation and malignant transformation (decreased apoptosis). H-Ras is a GTPase in the Ras family which has a role in various signal transduction pathways and is generally linked with cell membranes due to the presence of an isoprenyl group on its c-terminus [14-16]. Once H-Ras is activated (turned on) it recruits and activates proteins needed for the propagation of the receptor’s signal such as c-Raf. H-Ras binds to GTP in the active state and holds a fundamental enzymatic activity that cleaves the terminal phosphate of this nucleotide converting it to GDP, which ultimately turn off H-Ras.

Causative relations and interactions existing among survivin proteins and H-Ras will provide additional data to comprehend the possibility of designing effective anti-cancer drugs.

## BACKGROUND: RAS PATHWAY AND APOPTOTIC PROTEINS

Ras proteins are also essential for numerous signal pathways that control processes such as proliferation and apoptosis. Ras activates serine/threonine protein kinase Raf, which in turn activates another serine/threonine protein kinase MEK that triggers MAPK. Raf, MEK, and MAPK are considered mitogen-activated kinases. The deregulation of Ras which leads to increased metastasis (movement of cancerous cells) and decreased apoptosis often results in cancers. Since Ras leads to numerous tumors, then it would be valuable to discover a drug that is able to impose regulation into the Ras pathway, or eradicate cells with uncontrolled Ras pathways. Regulation of pro-apoptotic proteins under normal cell conditions of non-apoptotic cells is not understood entirely. As a result molecules that regulate apoptosis are being studied as potential targets for anti-cancer drug therapies. A MAPK pathway is shown in Fig. (**1**).

Since H-Ras is known to down-regulate survivin [17] and Delta-Ex3 has a role in angiogenesis [12], this work is then set to explore potential interactions between Delta-Ex3 and H-Ras in order to design apoptotic-based and angiogenesis-based anti-cancer drugs.

## COMPUTATIONAL METHODS: ROADMAP OF E-DRUG DESIGN AND DISCOVERY

Several software tools and “omic” databases are used to design a potential lead composed of a receptor and a ligand. Tools used in this work include Chimera [18], SimDOCK [19], PocketDepth [20], and e-HiTS [21]. Protein databases used in this study include PDB [22] and Swiss-Prot [23].

H-Ras and its ligands CAG and XY2 are shown in Fig. (**2**). CAG and XY2 appear clearly on top center and top left of Fig. (**2**). Delta-Ex3 is depicted in Fig. (**3**).

## SEARCHING FOR BINDING SITES, POCKETS ON DELTA-EX3 AND H-RAS

Several *In silico* experiments were conducted to search for binding sites, pockets and cavities on Delta-Ex3 and H-Ras. Fig. (**4**) depicted a pocket on H-Ras.

Various pockets were found on Delta-Ex3. Fig. (**5**) shows one pocket clearly visible in white.

The next experiments focused on studying the possibility of interacting Delta-Ex3 with ligands of H-Ras, i.e. CAG and XY2 shown in Figs. (**6**,**7**) respectively. The resulting complexes generated from the *In silico* experiments represent the expected hits for future anti-cancer drug investigation.

## DOCKING AND ANALYSIS

Molecular docking is used here to predict the conformation, orientation, and poses of the ligand XY2 within the active site of survivin Delta-Ex3 in order to form a stable complex. Knowing the preferred orientation could be used to predict the strength of association and/or binding affinity between XY2 and Delta-Ex3 using scoring functions. Chimera and e-HiTS tools are used to perform many of the following steps. The computational procedures were carried out on an HP computer with an Intel Pentium 4 processor, 2 GHz and 2 GB RAM.

1. Protein (“Receptor”) input file preparation: Using the survivin Delta-Ex3 protein structure (PDB code: 1E31B), a “clean input file” was generated by removing water molecules, ions, and subunits not involved in ligand binding from the original structure file. Subsequently, hydrogen atoms were added to the receptor and the active site was inspected to make suitable corrections of protonation states of charged residues. Local minimization was then performed in the presence to relieve potential bad contacts, at the same time maintaining the protein conformation as close as possible to that observed in the crystallographic model. The resulting receptor model was saved to a PDB file.
2. Ligand input file preparation: The ligand XY2 was removed from H-Ras protein (PDB code: 2EVW) and its structure was saved to a PDB file.
3. e-HiTS docking tool: 32 potential binding results were found when ligand XY2 was docked into Delta-Ex3. It took 4 minutes to complete the entire process and the best score was -3.247 which includes terms describing the inter- and intramolecular energies.

XY2 ligand is shown docked along Delta-Ex3’s surface and deeply embedded in a particular active site (Fig. **8**).

## CONCLUDING REMARKS AND FUTURE WORK

**Fig. (7):**
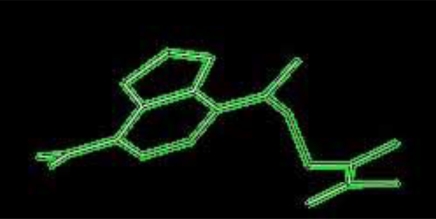
Ligand XY2 of H-Ras.

**Fig. (8):**
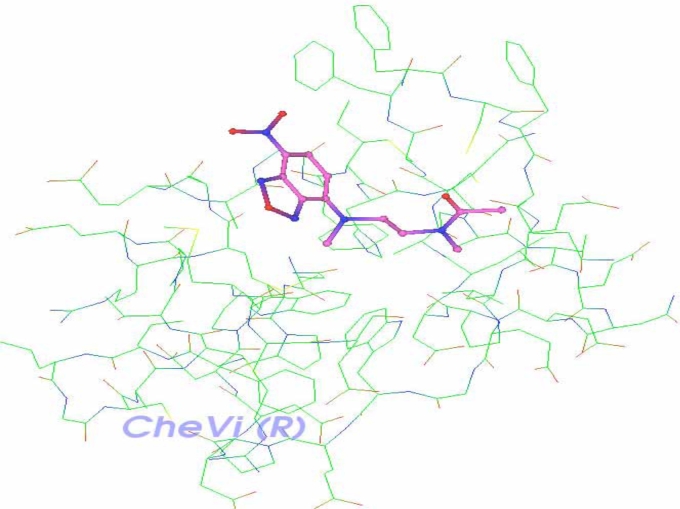
Complex receptor-ligand: Delta-Ex3 and XY2.

Caldas and his team [11] reported that Delta-Ex3 is found to have a role in angiogenesis, and consequently, this paper attempted to identify a possible cell signaling that induces apoptosis and inhibits angiogenesis through survivin isoform Delta-Ex3. In addition, H-Ras was found to down-regulate Delta-Ex3 [15], thus, potential interactions between a ligand of H-Ras and Delta-Ex3 are explored in this paper. Fig. 7 illustrates such possible interactions between Delta-Ex3 and HRas, where a binding of the HRas ligand XY2 is deeply embedded on Delta-Ex3’s surface. Additional lab experiments would elucidate the corresponding signaling process vis-à-vis apoptosis and angiogenesis.

The identified complex “Delta-Ex3 and XY2” shown in figure 7 serves as a protein complex that requires further *In* *Silico* processing including lead structure optimization. This information could assists researchers in designing drug compounds that bind selectively and tightly onto survivin Delta-Ex3..

The next phase of this project is to improve the design of survivin-based anti-cancer drug that induces apoptosis and inhibits tumor angiogenesis through the design of a multi-peptide link between XY2 and Delta-Ex3 [24-26].

## Figures and Tables

**Fig. (1):**
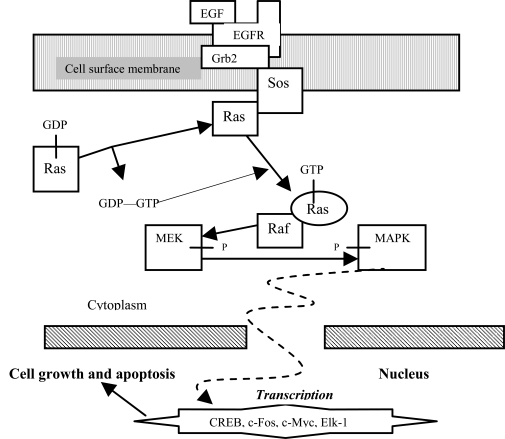
MAPK Pathway.

**Fig. (2):**
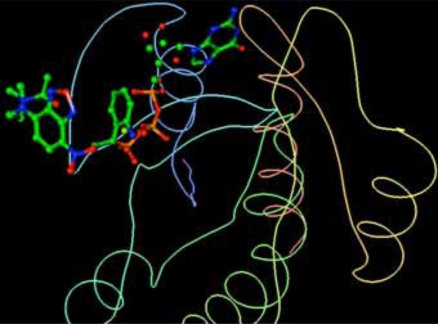
H-Ras and its ligands CAG and XY2.

**Fig. (3):**
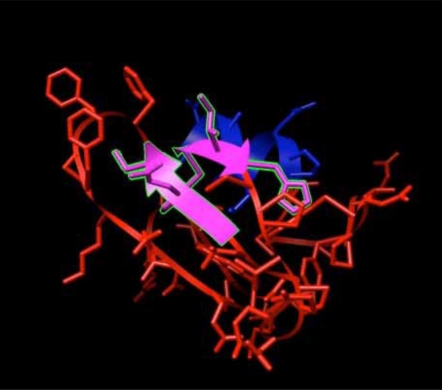
Delta-Ex3: Survivin protein without exon 3.

**Fig. (4):**
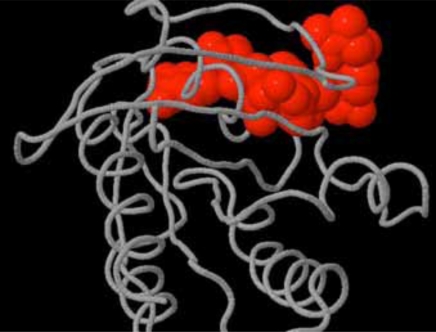
Pocket (shown in red) on H-Ras.

**Fig. (5):**
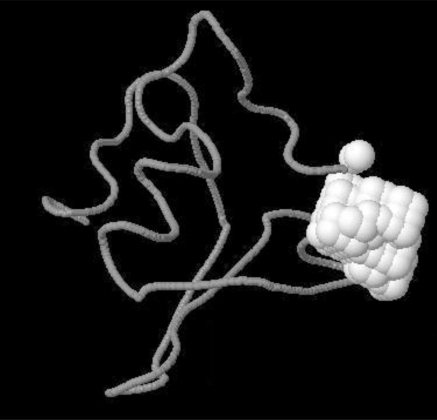
Pocket (shown in white) on Delta-Ex3.

**Fig. (6):**
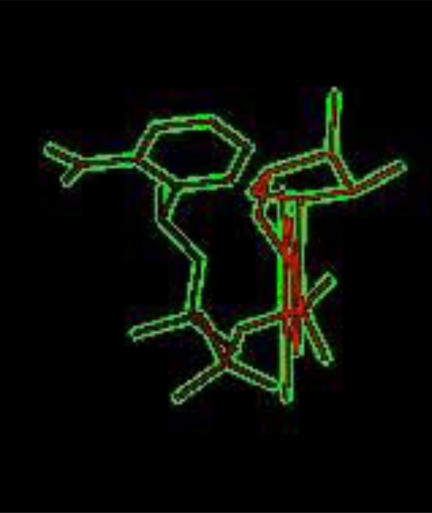
Ligand CAG of H-Ras.
